# Supplementary material for: Evaluation of a Live Attenuated S. sonnei Vaccine Strain in the Human Enteroid Model
Source: Pathogens. 2021 Aug 25;10(9):1079. doi: 10.3390/pathogens10091079 (PMC8468197; doi:10.3390/pathogens10091079)
Supplement: Supplementary file 1 [file pathogens-10-01079-s001.zip › pathogens-1248516-supplementary.pdf]

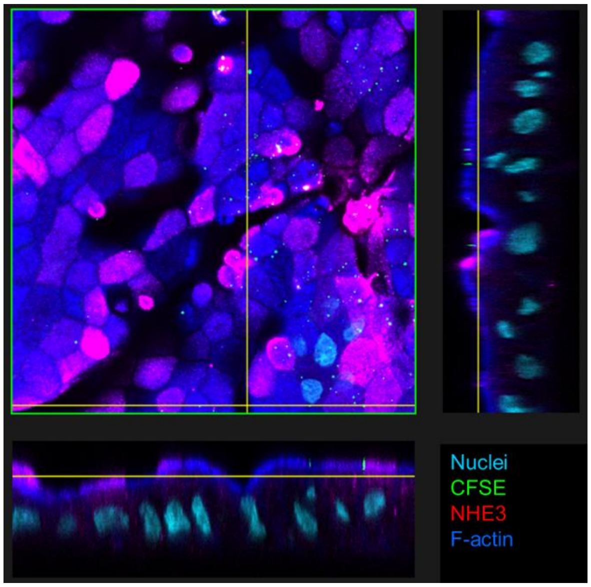

Supplementary Figure 1 (S1). Enteroid monolayers were stained with anti-F-actin to visualize the brush border (blue), anti-NHE3 to visualize the NHE3 transporter (red) and Hoechst to visualize nuclei (light blue). *S. sonnei* 1233-SP was staining with CFSE prior to invasion (green).

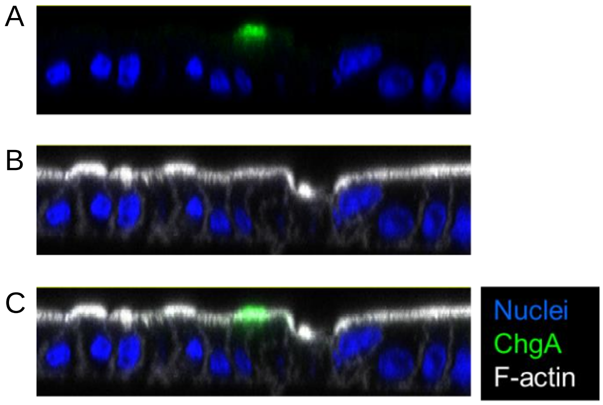

Supplementary Figure 2 (S2). Enteroid monolayers were stained with anti-chromagranin A (ChgA) for enteroendocrine cells (green) (A and C) and anti-F-actin to visualize the brush border (white) (B and C), and Hoechst to visualize nuclei (blue) (A, B, and C).
